# Metabolic Landscape and Cell-Type-Specific Transcriptional Signatures Associated with Dopamine Receptor Activation in the Honeybee Brain

**DOI:** 10.3390/biology15020174

**Published:** 2026-01-17

**Authors:** Miaoran Zhang, Kai Xu, Meng Xu, Jieluan Li, Yijia Xu, Qingsheng Niu, Xingan Li, Peng Chen

**Affiliations:** 1 Key Laboratory of Pathobiology, Ministry of Education, Jilin University, Changchun 130021, China; mrzhang20@mails.jlu.edu.cn; 2 Key Laboratory for Bee Genetics and Breeding, Jilin Provincial Institute of Apicultural Sciences, Jilin 132108, China; 3 Stroke Center, Department of Neurology, The First Hospital of Jilin University, Changchun 130021, China

**Keywords:** *Apis mellifera*, brain energy metabolism, dopamine, glycolysis, pentose phosphate pathway

## Abstract

Background: Honeybees sustain vital ecological roles through foraging behavior, which provides pollination services and is likely regulated by dopamine signaling coupled to brain energy metabolism. However, the genetic and metabolic mechanisms underlying this regulation remain unclear. Methods: We treated honeybee workers with the dopamine receptor agonist bromocriptine and employed an integrative approach, combining liquid chromatography–mass spectrometry (LC–MS) metabolomics with single-nucleus RNA sequencing (snRNA-seq). Results: Metabolomics revealed increased levels of N6-carboxymethyllysine (CML) and a coordinated shift in central carbon metabolites, including higher glucose, pyruvate, and lactate within glycolysis, and ribose-5-phosphate in the pentose phosphate pathway (PPP). Integration with transcriptomics showed heterogeneous responses: glial cells exhibited higher glycolysis pathway scores and upregulated hexokinase expression compared to neurons, whereas major PPP enzymes were upregulated in both glial and neuronal subsets. Conclusions: These findings suggest that dopamine receptor activation is associated with altered whole-brain metabolic profiles and concurrent, cell-type-specific upregulation of glycolytic and PPP enzyme genes, particularly in glia. This study characterizes these neuro-metabolic associations, offering insights into the cellular and metabolic basis of foraging behavior in worker bees.

## Simple Summary

The pollination services provided by honeybees make them vital for global food security. To perform demanding tasks such as foraging, bees must manage their energy efficiently, a process regulated by brain chemicals such as dopamine. However, it is not fully understood how dopamine specifically alters the brain’s energy usage. In this study, we treated honeybees with a substance that activates dopamine receptors and used advanced analysis to examine changes in their brain chemistry and gene activity. We found that dopamine receptor activation coincides with a shift in metabolism, causing the brain to consume more sugar for energy. Crucially, we observed that this energy boost occurs primarily in glial cells—the “support cells” that protect and feed the neurons—rather than in the nerve cells themselves. We also detected chemical markers indicating that this high-energy mode produces stress byproducts. These results suggest that support cells are metabolically active in fueling the honeybee brain during complex behaviors. Understanding these internal energy mechanisms provides new insights into how bees adapt to environmental stress, which is essential for understanding the metabolic constraints that support the demanding task of foraging.

## 1. Introduction

In honeybee colonies, workers flexibly allocate limited energy to sustain vital ecological tasks, particularly foraging and pollination, which is a process likely regulated by neuromodulatory systems [1,2,3,4,5]. Recent studies indicate that these behavioral states are not merely fueled by energy consumption but are tightly coupled to specific reconfigurations of brain energy metabolism in honeybees. For instance, highly aggressive individuals exhibit a shift toward aerobic glycolysis, characterized by increased glycolytic gene expression and metabolite levels alongside relatively reduced oxidative phosphorylation. Consistently, exposure to alarm pheromones, which elicit colony-level aggression, shifts brain metabolism toward a more glycolytic state [6,7]. These findings support a model in which behavioral specialization and responses to environmental challenges are intrinsically supported by the reprogramming of central carbon metabolism. However, while the correlation between behavior and metabolic state is well-documented, the upstream regulatory signals that orchestrate this metabolic switch remain to be identified.

Neuromodulatory systems, particularly biogenic amines, are the likely candidates for coupling environmental cues to such metabolic transition. Dopamine (DA) serves as a major central hub in this context: it may influence behavioral output and integrate social signals such as queen mandibular pheromone in higher-order brain regions [8,9,10,11,12,13,14]. Crucially, beyond its behavioral role, emerging evidence suggests that dopamine directly modulates the neuroendocrine control of glycolysis. In mice, elevated dopamine levels have been shown to increase phosphoglycerate kinase-1 (PGK1) activity and enhance glycolysis [15]; in *Drosophila melanogaster*, administration of L-DOPA reduces systemic glucose levels, suggesting that dopamine promotes glycolytic flux [16]. These cross-species lines of evidence imply that the dopamine system likely is integral to coupling social signals and behavioral state transitions with the reprogramming of brain energy metabolism. However, the precise molecular and cellular mechanisms by which dopamine drives these metabolic shifts in the honeybee brain remain unclear.

To trace dopamine-dependent shifts in brain energy metabolism at a molecular level, insights from mammalian studies offer a potential biochemical approach. Research has shown that advanced glycation end products (AGEs) such as N6-carboxymethyllysine (CML) accumulate under conditions of high glycolytic flux and oxidative or carbonyl stress [17,18,19]. CML is widely recognized as a stable biomarker of oxidative stress in metabolic and degenerative diseases [20,21,22]. However, beyond these pathological associations, CML accumulation fundamentally reflects the intensity of upstream carbon flux and dicarbonyl byproducts. Consequently, AGE-related metabolites could serve as integrative signatures of carbonyl and oxidative stress associated with dopamine-related metabolic reprogramming, yet it is unknown whether the activation of dopamine receptors in the insect brain is sufficient to drive such biochemical signatures.

Recent advances in single-cell and single-nucleus transcriptomics have provided insights into the cellular architecture and function of the brains of honey bees and other insects. These studies have not only revealed the diversity of cell populations such as Kenyon cells, glia, and optic lobe neurons, but have also linked specific cell types to caste differentiation, behavioral maturation, and social aggression [2,23,24]. Meta-analyses and single-cell work further suggest that glial populations, though numerically sparse in insect brains, may be involved in metabolic plasticity and behavioral phenotypes [25,26]. However, it remains unclear which honeybee brain cell types undergo metabolic shift in response to dopamine signaling, and whether these effects preferentially engage glycolytic and pentose phosphate pathway activity in specific neuronal or glial subsets. Therefore, combining single-cell-resolution transcriptomics with metabolic state analysis to decode the cellular mechanisms linking dopamine signaling and brain energy metabolism is an important objective.

In this study, we employed an integrative multi-omics approach, combining untargeted and targeted metabolomics with single-nucleus RNA sequencing (snRNA-seq) in dopamine receptor agonist-treated honeybee brains. This strategy enabled us to define dopamine-associated metabolic signatures at the whole-brain level and map these central carbon pathway shifts to specific cell types, providing a cell-type-resolved perspective on metabolic alteration in honeybee; such resolution is essential for understanding the bioenergetic mechanisms sustaining neural function during complex behaviors.

## 2. Materials and Methods

### 2.1. Drug Treatments

Drug treatments were delivered at the whole-colony level (≈15,000 workers per colony) using a fine mist applied directly onto the bees, a procedure previously established for pharmacological exposure in eusocial insects [27]. Bromocriptine (Gedeon Richter Plc., Budapest, Hungary), a dopamine receptor agonist, was dosed by scaling the maximal recommended human intake to the average worker bee mass (0.1 g, relative to a 60 kg human), yielding an estimated exposure of ~2 μg per bee (30 mg per colony) [28]. To account for genetic and environmental variability, the bees used in all experiments were sourced from three independent colonies maintained under identical conditions. The main experiment comprised two treatment conditions monitored over five consecutive days. Colonies in the bromocriptine (BRC) group received a daily spray of a solution containing twelve 2.5 mg bromocriptine tablets dissolved in 100 mL ddH_2_O, whereas the control colonies were sprayed with 100 mL ddH_2_O only. In all cases, solutions were evenly sprayed over the hive under standardized outdoor conditions to ensure comparable exposure across individuals [27].

### 2.2. Sample Collection and Brain Dissection

The honeybees used in this study were obtained from colonies raised at the Jilin Academy of Apiculture Science (Jilin City, Jilin Province, China). The colonies were maintained outdoors under natural environmental conditions, and all specimens were of the species *Apis mellifera carnica*. Forager bees were collected at hive entrances between 9:00 and 16:00 while carrying pollen baskets and returning to the nest. Collected bees were anesthetized on ice in a dissection dish and washed twice with ethanol and phosphate-buffered saline (PBS; Thermo Fisher, Cat# 10010023, Waltham, MA, USA). Brains were dissected in PBS on ice under a stereomicroscope (OLYMPUS EP50, Olympus Corporation, Tokyo, Japan). The surrounding tracheae, ocelli, and compound eyes were carefully removed, and the brains were rinsed with 1 mL of PBS.

### 2.3. Quantitative PCR

To validate activation of dopamine receptor signaling prior to metabolomic profiling, we measured the transcript levels of the D2-like dopamine receptor gene *Dop3* and the downstream effector gene *PKA-c1* using quantitative PCR (qPCR). For each treatment condition (BRC and ddH_2_O), five independent biological replicates were collected. These replicates were sourced across the three independent colonies to ensure representative sampling. Each replicate consisted of pooled brains from eight forager honeybees (workers returning to the hive with pollen loads on the hind legs).

Total RNA was isolated using a commercial RNA extraction kit (Sangon Biotech, Cat# B511321, Shanghai, China) following the manufacturer’s instructions. In brief, dissected brains were homogenized in TRIzol reagent (Invitrogen, Cat# 15596026, Carlsbad, CA, USA), followed by phase separation with chloroform (Sigma-Aldrich, Cat# C2432, St. Louis, MO, USA). The aqueous phase was combined with anhydrous ethanol and passed through the silica-based columns supplied with the kit. After a series of washing and centrifugation steps, RNA was eluted in 30 µL of DEPC-treated water and stored at −80 °C until use.

First-strand cDNA was synthesized from total RNA using a reverse transcription kit (BBI, Cat# B639251, Shanghai, China). Reactions were assembled according to the manufacturer’s recommendations and run on a thermal cycler with the following program: 25 °C for 5 min, 42 °C for 30 min, and 85 °C for 5 min to inactivate the reverse transcriptase.

qPCR reactions were prepared with the GoTaq qPCR kit (Promega, Cat# A6001, Madison, WI, USA) and run on an Applied Biosystems 7500 real-time PCR system (Foster City, CA, USA). Primer sequences for *Dop3*, *PKA-c1*, and the reference gene *GAPDH* are provided in Table S1. Each reaction was performed in technical duplicate using the cycling conditions recommended for the GoTaq kit: initial activation at 95 °C for 2 min, followed by 40 cycles of 95 °C for 15 s and 60 °C for 1 min. A dissociation curve analysis was included at the end of the run to verify amplification specificity.

Relative expression levels were calculated using the 2^−ΔΔCt^ method with *GAPDH* as the internal control. For each gene, fold changes between the BRC and ddH_2_O groups were computed from five biological replicates per condition. Group differences were assessed using unpaired, two-tailed Student’s *t*-tests implemented in R (v4.4.0), and *p* < 0.05 was considered statistically significant.

### 2.4. Untargeted Metabolomics

For untargeted metabolomics, six biological replicates were generated for each treatment condition, with each colony contributing two replicates to capture inter-colony variance. Each replicate comprised pooled brain tissue (~15 mg) collected from 10 worker foragers per group. Metabolites were extracted by grinding tissue in liquid nitrogen and suspending the powder in 500 µL of ice-cold 80% methanol. After incubation on ice for 5 min, samples were centrifuged at 15,000× *g* for 20 min at 4 °C. The supernatant was diluted with LC–MS-grade water to a final methanol content of 53% and centrifuged again; the resulting clarified extract was used for UHPLC–MS/MS profiling.

Chromatographic separation was carried out on a Vanquish UHPLC system (Thermo Fisher, Waltham, MA, USA) coupled to an Orbitrap Q Exactive HF mass spectrometer (Thermo Fisher, Waltham, MA, USA) using a Hypersil Gold column (100 × 2.1 mm, 1.9 µm) with a 12 min gradient at 0.2 mL/min. Data were acquired in both positive and negative ionization modes using water/methanol mobile phases containing either 0.1% formic acid (positive mode) or 5 mM ammonium acetate at pH 9.0 (negative mode). Quality control (QC) samples were prepared by pooling equal aliquots from all study samples and injected at regular intervals throughout the analytical sequence to monitor instrument performance.

Raw files were processed in Compound Discoverer 3.3 (Thermo Fisher, Waltham, MA, USA) for peak detection, retention time alignment, and intensity extraction. Metabolites were annotated by matching spectral features to mzCloud, mzVault, and in-house MassList libraries with a 5 ppm mass tolerance on precursor ions and MS/MS spectra; only features with full MS1/MS2 matches were retained. Peak intensities were normalized to total ion signal, and features showing a coefficient of variation > 30% in pooled QC samples were removed. Subsequent data analysis was performed in R (v4.4.0). Data cleaning and reshaping were conducted using the tidyverse (v2.0.0) suite of packages (including dplyr, tidyr, and readr) [29]. Visualizations, including PCA score plots and volcano plots, were generated using ggplot2 (v4.0.0) [30]. Orthogonal partial least squares discriminant analysis (OPLS-DA) models were fitted to discriminate BRC and ddH_2_O groups. Model quality was evaluated based on the cumulative model fit (R^2^Y) and predictive ability (Q^2^Y). To further evaluate the contribution of individual metabolites to the group separation, the correlation coefficients between the metabolite abundance and the predictive component of the OPLS-DA model were calculated using the ropls R package (v1.40.0). To assess the risk of overfitting, a permutation test with 200 iterations was performed to generate empirical *p*-values for the class discrimination. Variable importance in projection (VIP) scores were extracted. For each metabolite, group differences were assessed using two-sided Student’s *t*-tests; the resulting *p*-values were adjusted for multiple testing using the Benjamini–Hochberg false discovery rate (FDR) procedure. Differential metabolites were defined as those with VIP > 1, FDR-adjusted *p* < 0.05, and a log_2_ fold change ≥ 0.8 or ≤−0.8. The same normalization and statistical framework were applied to all annotated metabolites.

### 2.5. Targeted Metabolomics Analysis

Brain samples were handled on ice throughout preparation to limit degradation. For each measurement, brains from 10 individual bees were pooled to obtain ~15 mg wet tissue, allowing for minor variation in individual brain mass. A total of six biological replicates per treatment group were analyzed, ensuring balanced representation from the three source colonies. Each pooled sample was weighed, combined with 20 µL deionized water and 10 ceramic beads, and homogenized for 3 min in a Bullet Blender (BB24, Next Advance Inc., Troy, NY, USA). A 120 µL aliquot of ice-cold methanol containing a mixture of stable isotope-labeled internal standards (Glutamic acid-^13^C_5_, Pyruvic acid-^13^C_3_, Succinic acid-d_4_, Malic acid-d_3_, Aspartic acid-d_3_, Lactic acid-^13^C_3_, Serine-d_3_, Glutamine-^13^C_5_, D-Glucose-d_7_, Fructose-^13^C_6_, Citric acid-d_4_, α-Ketoglutaric acid-^13^C_5_, and Fructose-6-phosphate-^13^C_6_) was then added, followed by a second 3 min homogenization. Samples were centrifuged at 18,000× *g* for 15 min at 4 °C (Microfuge 20R, Beckman Coulter, Indianapolis, IN, USA), and 20 µL of the resulting supernatant was dispensed into each well of a 96-well plate.

For derivatization, 20 µL of 200 mM 3-nitrophenylhydrazine and 20 µL of 120 mM EDC hydrochloride were added to each well and incubated at 30 °C and 1450 rpm for 60 min (MSC-100, Hangzhou Allsheng Instruments Co., Ltd., Hangzhou, China). The reaction was stopped by adding 350 µL of ice-cold methanol solution. After centrifugation at 4000× *g* for 20 min at 4 °C (Allegra X-15R, Beckman Coulter, Indianapolis, IN, USA), 150 µL of the clear supernatant was transferred to a fresh 96-well plate for LC–MS analysis.

Targeted metabolite profiling was carried out on an Acquity I-Class UPLC system (Waters Corporation, Milford, MA, USA) coupled to a Xevo TQ-S mass spectrometer (Waters Corp., Milford, MA, USA). Injection order was randomized within and across treatment groups, with QC and blank samples periodically injected to monitor analytical stability. Raw UPLC–MS/MS data were processed in MassLynx v4.1 (Waters) for peak detection, integration, calibration, and quantification. Statistical analyses were performed in R v4.4.0. Differential metabolite abundance was determined using the limma package (v3.48.0) [31], which employs linear models and empirical Bayes statistics. Hierarchical clustering heatmaps were generated using the ComplexHeatmap package (v2.27.0) [32] with color mapping provided by circlize (v0.4.17) [33]. Pathway enrichment analysis was conducted using the Small Molecule Pathway Database (SMPDB) [34] and the MetaboAnalyst online platform (v5.0) [35].

### 2.6. Library Preparation, Processing, and Preliminary Analyses of snRNA-Seq Data

For snRNA-seq, single nuclei were isolated using a mechanical extraction protocol optimized for honeybee brain tissue [36]. Briefly, dissected brains were homogenized in 2 mL of ice-cold nuclei isolation buffer containing 10 mM Tris-HCl, 146 mM NaCl, 1 mM CaCl_2_, 21 mM MgCl_2_, 0.1% NP-40, and RNase inhibitor using a Dounce homogenizer. The homogenate was filtered through a 40 μm cell strainer to remove debris and centrifuged at 500× *g* for 5 min at 4 °C. The supernatant was discarded, and the nuclear pellet was resuspended in wash buffer. Prior to library preparation, nuclei quality and concentration were assessed using DAPI staining to verify structural integrity and absence of clumping. A single sequencing library was generated for each treatment condition (BRC or ddH_2_O). To maximize biological representativeness and mitigate colony-specific effects, each library was prepared by pooling 30 forager brains, with 10 brains sampled from each of the three independent colonies. Nuclei isolation and library construction were then performed. Sequencing libraries were generated using the Chromium Single Cell 3′ Library and Gel Bead Kit v3.1 (10×Genomics, Pleasanton, CA, USA) and sequenced on an Illumina NovaSeq 6000 platform (San Diego, CA, USA) with a paired-end 150 bp (PE150) strategy, targeting at least 100,000 reads per nucleus.

CellRanger (v7.0.1, 10× Genomics) was used to demultiplex raw reads, align them to the *Apis mellifera* Amel_HAv3.1 reference genome, and generate gene–barcode count matrices. The resulting matrices for the BRC and ddH_2_O groups were imported into Seurat (v5.0.0) [37] to create one Seurat object per group. Quality control filters were applied at the nucleus level using the following criteria: log_10_GenesPerUMI > 0.85, 200 < nFeature_RNA < 5000, and mitochondrial gene percentage (percent.mt) < 5%. The log10GenesPerUMI metric was computed as log_10_(nFeature_RNA)/log_10_(nCount_RNA) to capture library complexity. Potential doublets were identified and removed using the scDblFinder R package (v1.11.4) with dbr.sd = 1.

After QC and doublet removal, the two Seurat objects were integrated using the canonical correlation analysis (CCA)-based integration workflow implemented in Seurat. Standard preprocessing steps (NormalizeData, FindVariableFeatures, ScaleData) were applied, followed by principal component analysis (PCA). Unsupervised clustering was performed in the shared low-dimensional space using the first 50 principal components, and clusters were visualized with Uniform Manifold Approximation and Projection (UMAP). Detailed parameter settings for integration, dimensionality reduction, and clustering are provided in the accompanying analysis code. Cell types were manually annotated based on the expression patterns of established canonical marker genes for honeybee brain populations (listed in Table S2). Annotation was performed by visualizing the expression distribution of these markers using dot plots. A cluster was assigned to a specific cell type identity if it exhibited a distinct enrichment profile for the corresponding markers, characterized by higher average expression levels and a greater proportion of expressing nuclei compared to other clusters.

### 2.7. Cell Type Proportion Analysis

To test whether dopamine receptor activation was associated with shifts in cell type composition, we compared the proportions of major cell types between BRC and ddH_2_O groups using the scProportionTest (v1.0) R package [38]. For each annotated cluster, the proportion of nuclei assigned to that cell type was calculated within each sample, and differences in proportions between treatment conditions were evaluated using the permutation-based framework implemented in scProportionTest. Briefly, the null distribution of proportion differences was generated by repeatedly permuting the condition labels across nuclei and recalculating cluster-wise proportions, and empirical *p*-values were derived from these permutation tests. Confidence intervals for the magnitude of proportion differences were obtained via bootstrapping, as provided by the package. Resulting *p*-values were adjusted for multiple testing using the Benjamini–Hochberg procedure, and clusters with FDR-adjusted *p* < 0.05 were considered to show significant changes in cell type proportions.

### 2.8. Gene Set Definition and JASMINE Scoring

Gene sets for glycolysis, gluconeogenesis, the pentose phosphate pathway (PPP), insulin/insulin-like growth factor signaling (IIS), and the tricarboxylic acid (TCA) cycle were derived from Gene Ontology (GO) biological process annotations and mapped to *Apis mellifera* orthologs using available homology information. For each GO-based gene set, we first summarized per-gene detectability across cell types by calculating (i) the maximum percentage of nuclei expressing the gene (max_pct) and (ii) the maximum average expression (max_avg) across annotated cell types. To restrict scoring to genes that were robustly detected, gene-specific thresholds were defined within each gene set as the 70th percentile of max_pct (with a lower bound of 5%) and the 70th percentile of max_avg; genes exceeding both thresholds were retained. To avoid over-filtering, if fewer than five genes passed these criteria, the top-ranked genes based on a composite score combining scaled max_pct and max_avg (equal weights) were retained to a minimum of five genes per gene set.

Pathway expression scores at the single-nucleus level were computed using the JASMINE algorithm for single-cell signature scoring [39]. For each gene set and each nucleus, JASMINE computes two complementary metrics: an approximate mean rank of the signature genes among all expressed genes in that cell, and an enrichment score reflecting the overrepresentation of the signature within the expressed gene set. Both components were scaled to the range 0–1 and averaged to yield a composite activity score per nucleus for each gene set. These single-nucleus scores were then used to compare the distribution of pathway activity across cell types.

### 2.9. Differential Expression Analysis

Differential expression analysis between BRC and ddH_2_O conditions was performed separately within each annotated cell type using standard functions implemented in Seurat. Prior to testing, gene expression counts were normalized using Seurat’s Normalize Data function and log_10_-transformed according to the package defaults. For each gene and each cell type, nuclei were partitioned by treatment group, and group-wise differences were assessed using non-parametric tests embedded in the Seurat differential expression framework. Resulting *p*-values were adjusted for multiple comparisons using the Benjamini–Hochberg FDR procedure, and genes with FDR-adjusted *p* < 0.05 were considered significantly differentially expressed. log_2_ fold changes reported in the main text are defined as log_2_(BRC/ddH_2_O) based on normalized expression values within the corresponding cell type.

To systematically quantify the magnitude of transcriptional perturbation and cell type responsiveness, we employed two complementary computational approaches. The Augur R package (v1.0.0) [40] was used to prioritize cell populations by calculating Area Under the Curve (AUC) scores, reflecting the separability of treatment conditions within each cell type. Additionally, the scDist R package (v1.1.5) [41] was utilized to compute statistical distances, providing a robust metric for the intensity of transcriptional remodeling between BRC and control groups.

Functional interpretation of the identified signatures was performed using g:Profiler (2025 update). For this analysis, differentially expressed genes were selected based on an FDR-adjusted *p* < 0.05, a log_2_ fold change (logFC) > 1.5, and a minimum expression percentage (min.pct) > 0.1. The organism was set to *Apis mellifera* (Western honeybee), and significant pathways were identified using the Bonferroni correction for multiple testing.

While 30 brains were pooled per condition to ensure biological diversity, single sequencing libraries were generated for each group. Statistical analyses were thus performed at the single-nucleus level to characterize cellular and metabolic state differences, as opposed to making population-level inferences. Accordingly, the reported significance values indicate differences in gene expression distributions across the recovered nuclei.

## 3. Results

**Figure 1:**
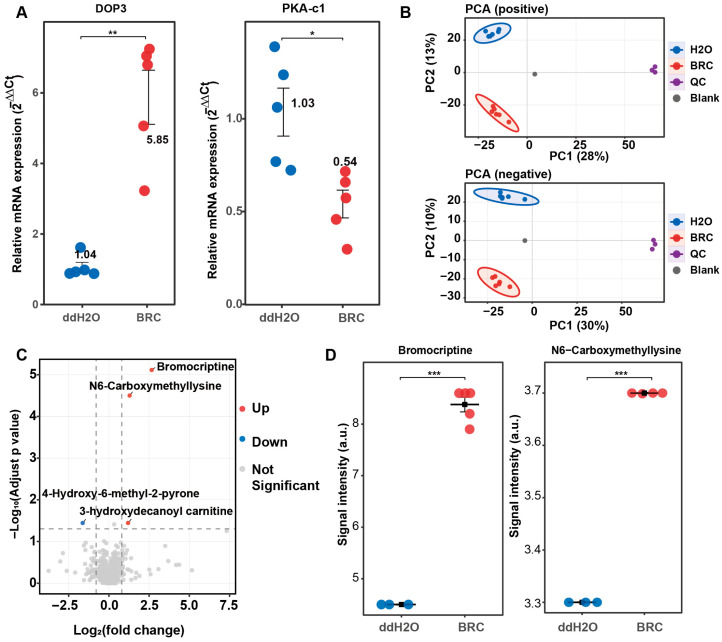
Validation in dopamine receptor activation and global metabolomic profiling of honeybee brains under bromocriptine treatment. (**A**) qPCR validation of the pharmacological model. The relative mRNA expression levels (2^−ΔΔCt^) of the D2-like dopamine receptor gene *Dop3* (**left**) and its downstream effector *PKA-c1* (**right**) are shown for the control (ddH_2_O, blue) and bromocriptine-treated (BRC, red) groups. Data represent mean ± SEM. Statistical significance was determined via a *t*-test (* *p* < 0.05; ** *p* < 0.01). (**B**) Principal component analysis (PCA) score plots of untargeted metabolomic data. The top panel shows positive ion mode (PC1 28%, PC2 13%) and the bottom panel shows negative ion mode (PC1 30%, PC2 10%). Samples are color-coded: ddH_2_O (blue), BRC (red), quality control (QC, purple), and blank (gray). Ellipses indicate 95% confidence intervals. (**C**) Volcano plot of differential metabolites in the BRC group versus ddH_2_O. The *x*-axis represents log_2_(fold change) and the *y*-axis represents −log_10_(adjusted *p*-value). Red dots indicate significantly upregulated metabolites, blue dots indicate downregulated metabolites, and gray dots represent non-significant changes. (**D**) Quantitative signal intensity analysis of key differential metabolites. Box plots show the signal intensity (a.u.) for bromocriptine (**left**) and N6-carboxymethyllysine (**right**). The BRC group (red) shows significantly higher levels compared to the ddH_2_O group (blue). *** *p* < 0.001.

To investigate the regulatory role of dopamine signaling in energy metabolism in honeybee brains, we established a dopamine receptor agonist (BRC) treatment model. The efficacy of the pharmacological manipulation was validated via qPCR before metabolomic analysis. Compared with the control group (ddH_2_O), the relative mRNA expression of the D2-like dopamine receptor gene *Dop3* was higher in the BRC-treated group (*p* < 0.01), whereas expression of the downstream effector gene protein kinase A catalytic subunit 1 (*PKA-c1*) was lower (*p* < 0.05) (Figure 1A). These changes in gene expression indicated activation of the dopamine receptor signaling pathway.

### 3.1. Bromocriptine Activation of D2-like Receptors Induces CML Accumulation

Untargeted metabolomic profiling was then performed to assess global metabolic changes induced by BRC treatment. In total, 870 metabolites were matched across positive and negative ion modes (Table S3). Principal component analysis (PCA) showed separation between BRC-treated and control brains in both ion modes, and quality control (QC) samples clustered tightly (Figure 1B). Differential metabolite analysis identified multiple metabolites with altered abundance between groups (Figure 1C). The exogenous compound BRC was detected at high abundance in the treatment group. N6-carboxymethyllysine and 3-hydroxydecanoyl carnitine were increased in the BRC group, whereas 4-hydroxy-6-methyl-2-pyrone was decreased (Figure S1A,B). Signal intensities of bromocriptine and N6-carboxymethyllysine were higher in the BRC group than in the controls (bromocriptine: log_2_FC = 0.95; N6-carboxymethyllysine: log_2_FC = 0.32; FDR-adjusted *p* < 0.001 for both) (Figure 1D).

### 3.2. Targeted Energy Metabolism Profiling Reveals a Dopamine-Associated Shift in Glycolysis, PPP, and TCA Intermediates

**Figure 2:**
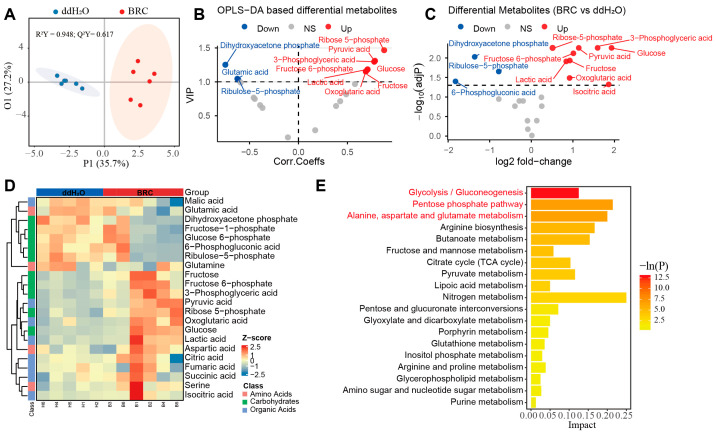
Targeted metabolomic profiling of energy metabolism pathways. (**A**) Orthogonal partial least squares discriminant analysis (OPLS-DA) score plot. The *x*-axis (P1) represents the predictive component, and the *y*-axis (O1) represents the orthogonal component, with the percentage of explained variance shown in parentheses. Colors distinguish the experimental groups (ddH2O: blue; BRC: red), and shaded areas represent confidence regions. OPLS-DA score plots showing separation between BRC and control groups (R^2^Y = 0.948; Q^2^Y = 0.617; permutation test *p* < 0.05). (**B**) Variable importance in projection (VIP) derived from the OPLS-DA model. The *x*-axis displays the correlation coefficients (Corr. Coeffs), and the *y*-axis displays the VIP scores. Points are colored according to their differential status (red: upregulated; blue: downregulated; gray: non-significant). (**C**) Volcano plot of targeted energy metabolites. The *x*-axis represents the log_2_ fold change, and the *y*-axis represents the −log_10_(adjusted *p*-value). Red and blue points denote upregulated and downregulated metabolites, respectively, while gray points indicate no significant difference. (**D**) Hierarchical clustering heatmap of differential metabolites. Rows correspond to individual metabolites, and columns correspond to samples (top annotation bars: blue for ddH_2_O, red for BRC). The color scale represents the row-scaled Z-score of metabolite abundance (purple: low abundance; orange: high abundance). The dendrogram on the left illustrates the clustering hierarchy of the metabolites. (**E**) Bar chart of KEGG pathway enrichment analysis. The *x*-axis represents the pathway Impact score, and the *y*-axis lists the enriched metabolic pathways. The color gradient of the bars corresponds to the statistical significance level −ln(P). The legend indicates the classification of metabolic pathways (amino acids, carbohydrates, organic acids).

To validate the accumulation of N6-carboxymethyllysine identified in the untargeted analysis and trace its potential upstream metabolic sources, we next performed targeted profiling of metabolites in glycolysis, the pentose phosphate pathway, and the TCA cycle. An orthogonal partial least squares discriminant analysis (OPLS-DA) model showed that metabolic profiles of the BRC and ddH_2_O groups were separated along the predictive component (P1 = 35.7%), indicating a treatment-associated metabolic shift, whereas the orthogonal component (O1 = 27.2%) captured variation not related to treatment (Figure 2A). In the OPLS-DA VIP volcano plot, metabolites with VIP > 1 and positive correlations with the predictive component—ribose-5-phosphate (R5P), glucose (Glc), fructose-6-phosphate (F6P), fructose (Fru), 3-phosphoglyceric acid (3-PG), pyruvic acid (Pyr), lactic acid (Lac), and oxoglutaric acid (2-OG)—were enriched in the BRC group relative to ddH_2_O (FDR-adjusted *p* ≤ 0.05), whereas dihydroxyacetone phosphate (DHAP), glutamate (Glu), and ribulose-5-phosphate (Ru5P) showed negative correlations (Figure 2B, Table S4).

Direct comparison of metabolite levels confirmed higher concentrations of several glycolytic intermediates and substrates in BRC-treated bees, including Pyr, Lac, 3-PG, glucose, and fructose. The tricarboxylic acid (TCA) cycle intermediate 2-OG was also increased. Within the pentose phosphate pathway (PPP), R5P and F6P were higher in the BRC group. In contrast, 6-phosphogluconic acid, DHAP, and Ru5P were lower in BRC-treated brains (Figure 2C and Figure S1C–M, Table S5). Furthermore, the hierarchical clustering heatmap of differential metabolites visually demonstrated a clear separation in metabolic profiles between the BRC and control groups, revealing a coordinated shift in key energy metabolites, including increased glycolytic and TCA cycle intermediates, following dopamine receptor activation (Figure 2D). Pathway enrichment analysis indicated that the differential metabolites were mainly assigned to glycolysis/gluconeogenesis, the pentose phosphate pathway, and alanine, aspartate and glutamate metabolism (*p* < 0.05) (Figure 2E, Table S6).

### 3.3. snRNA-Seq Reveals Cell-Type-Specific Changes in Enzyme Expression, with Prominent Effects in Glial Populations

**Figure 3:**
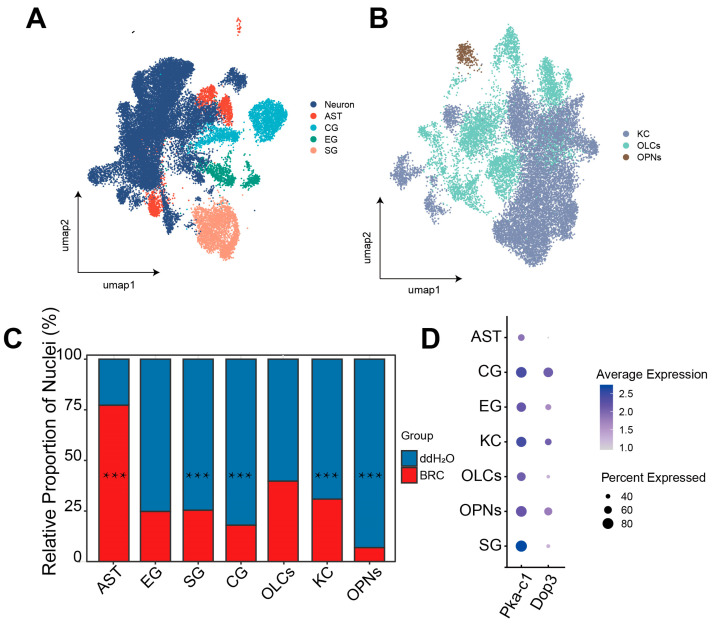
snRNA-seq analysis of cell-type-specific transcriptomic data and the distribution of dopamine pathways. (**A**) UMAP plot visualizing the major cell types identified in the honeybee brain. Each dot represents a single nucleus, colored by broad cell class annotations: Neuron, Astrocyte-like Glia (AST), Cortex Glia (CG), Ensheathing Glia (EG), and Surface Glia (SG). (**B**) UMAP plot showing the sub-clustering of neuronal populations. Colors distinguish specific neuronal subtypes: Kenyon Cells (KCs), Optic Lobe Cells (OLCs), and Olfactory Projection Neurons (OPNs). (**C**) Relative contribution of experimental groups to each identified cell cluster. The *X*-axis displays cell types ordered from glial populations (AST, EG, SG, CG) to neuronal populations (OLCs, KC, OPNs). The *Y*-axis represents the percentage of nuclei originating from either the BRC (treatment) or H_2_O (control) group within each specific cell type cluster (normalized to 100% for each cluster). This highlights the shift in group composition for each cell identity. Differences were assessed using scProportionTest (*** FDR-adjusted *p* < 0.001). (**D**) Dot plot of dopamine signaling marker gene expression. The *x*-axis represents cell types. Dot size indicates the percentage of nuclei expressing the gene (% detected), and color intensity represents the average scaled expression level.

To identify the specific cellular populations driving the observed systemic upregulation of glycolytic and pentose phosphate pathways, we performed single-nucleus transcriptomic analysis, initially characterizing cell-type-specific compositional shifts and dopamine receptor distributions. Analysis of 34,232 high-quality nuclei identified major neuronal (KCs, OLCs, OPNs) and glial (SG, CG, EG, AST) populations (Figure 3A,B and Figure S2A,B). Using the scProportionTest algorithm, we quantified differences in the relative abundance of recovered nuclei between BRC and control datasets. We found that the BRC group exhibited lower recovered proportions of OPNs, CG, SG, and KCs and a higher proportion of AST (Figure 3C, Table S7). We then mapped *Dop3* and *PKA-c1* expression across cell types. *Dop3* was mainly expressed in KCs, CG, and OPNs, whereas *PKA-c1* showed broad expression across clusters (Figure 3D).

**Figure 4:**
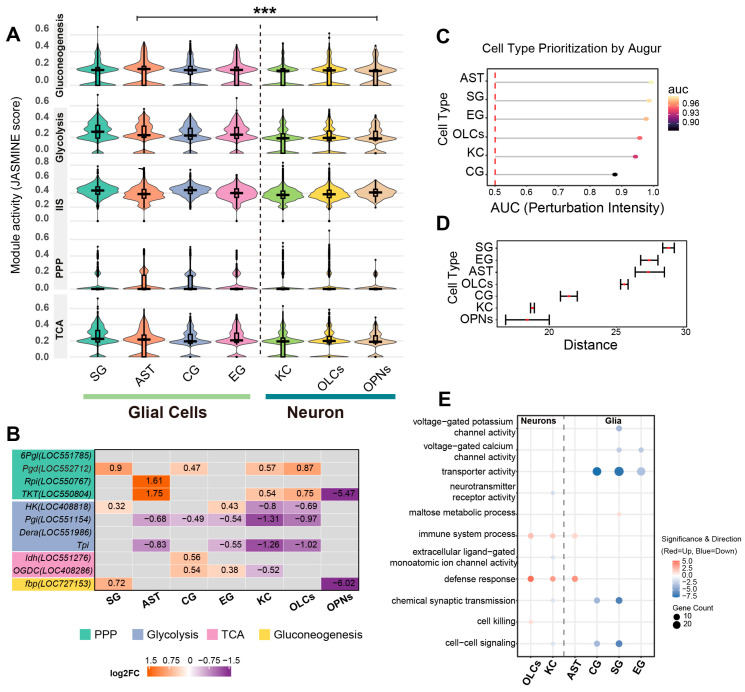
Cell-type-specific metabolic pathway activity and enzymatic transcriptional signatures. (**A**) Distribution of metabolic pathway module scores across cell types. Violin plots visualize the density and distribution of JASMINE module scores for key metabolic pathways (gluconeogenesis, glycolysis, IIS, PPP, and TCA cycle) across seven major cell clusters. Internal boxplots represent the statistical summary: the center line indicates the median, box limits define the interquartile range (IQR), and whiskers extend to 1.5× IQR. Statistical comparisons were performed between the aggregate glial population (SG, AST, CG, EG) and the neuronal population (KCs, OLCs, OPNs) using a two-sided Wilcoxon rank-sum test. Asterisks denote significant shifts in score distributions consistently observed across all examined metabolic pathways. (*** *p* < 0.001). (**B**) Heatmap profiling the transcriptional changes in rate-limiting metabolic enzymes. The color gradient represents the log_2_ fold change (log_2_FC) in BRC-treated nuclei relative to controls (orange: upregulated; purple: downregulated; gray: non-significant or low expression). Numerical values within cells indicate the specific log_2_FC. Gene names are color-coded by metabolic pathway: pentose phosphate pathway (green), glycolysis (purple), TCA cycle (pink), and gluconeogenesis (yellow). (**C**) Quantification of transcriptional perturbation intensity by Augur (v1.0.0). The dot plot displays the Area Under the Curve (AUC) for each cell type, serving as a metric for the magnitude of the transcriptional response to BRC treatment. Higher AUC values indicate greater responsiveness. (**D**) Statistical distance analysis using scDist (v1.1.5). The plot shows the estimated mixed-effects distance (dimensionless units in PC space) between BRC and control conditions for each cell type. Error bars represent 95% confidence intervals. (**E**) Functional enrichment divergence between neurons and glia. The dot plot compares representative enriched Gene Ontology (GO) terms highlighting the functional distinction across cell types. Dot size corresponds to the number of significant genes, and the color scale indicates the direction and significance of regulation (red: upregulated; blue: downregulated).

We next examined cell-type-specific metabolic reprogramming by integrating global pathway expression scoring with the transcriptional profiles of key rate-limiting enzymes. JASMINE module scores were calculated for each nucleus and summarized by cell type. As visualized in Figure 4A, we analyzed the distribution of pathway expression scores across cell populations to characterize metabolic heterogeneity. Overall, statistical comparisons between the aggregate glial and neuronal populations revealed significant differences across all examined metabolic pathways (*p* < 0.001), yet the specific distribution patterns varied distinctively by pathway. For glycolysis, glial clusters displayed a distribution density shifted towards higher values compared to neurons; notably, SG cells exhibited a right-skewed pattern with a tail extending into the upper score range, whereas neuronal clusters (KCs, OLCs) were distributed within a lower range. Regarding the PPP, AST displayed a wider interquartile range, distinguishing them from neuronal clusters which remained at baseline levels. Conversely, although statistically distinct in the aggregate comparison, the distributions for TCA cycle and IIS scores were broad and overlapped considerably across major cell types.

We further evaluated whether enzymes associated with the differential metabolites exhibited concordant transcriptional signatures by analyzing their expression patterns at the single-nucleus level to characterize cellular metabolic state differences across major neuronal and glial populations (Figure 4B, Table S8). For PPP genes, 6-phosphogluconate dehydrogenase (*Pgd*; *LOC552712*) was upregulated in several glial and neuronal populations in the BRC group, with positive log_2_FC values in SG, CG, KCs, and OLCs. Ribose-5-phosphate isomerase (*Rpi*; *LOC550767*) was increased in AST, and transketolase (*TKT*; *LOC550804*) was increased in AST, KCs, and OLCs, but decreased in OPNs. In the glycolysis/gluconeogenesis set, hexokinase (*HK*; *LOC408818*) was higher in SG and EG but lower in KCs and OLCs. Glucose-6-phosphate isomerase (*Pgi*; *LOC551154*) showed negative log_2_FC values in AST, CG, EG, KCs, and OLCs. Deoxyribose-phosphate aldolase (*Dera*; *LOC551986*), which links deoxyribose-phosphate metabolism to the glyceraldehyde-3-phosphate pool, was downregulated in AST, CG, EG, and KCs. Triosephosphate isomerase (*Tpi*) showed a similar pattern, with reduced expression in AST, EG, KCs, and OLCs. Within the gluconeogenic branch, fructose-1,6-bisphosphatase (*fbp*; *LOC727153*) was increased in SG and decreased in OPNs. For TCA-related genes, cytosolic isocitrate dehydrogenase (*Idh*; *LOC551276*) was upregulated in CG, and oxoglutarate dehydrogenase (*OGDC*; *LOC408286*) was upregulated in CG and EG but downregulated in KCs. These results indicate that the metabolite changes observed after BRC treatment are accompanied by cell-type-specific transcriptional signatures of key enzymes in glycolysis, the PPP, gluconeogenesis, and the TCA cycle.

To systematically quantify the magnitude of transcriptional perturbation across cell types, we evaluated the global responsiveness of each population. Cell type prioritization analysis identified AST and SG as the most sensitive populations, exhibiting the highest AUC scores (AUC > 0.90) (Figure 4C). This observation was consistently corroborated by statistical distance analysis, which revealed that glial populations (SG, EG, AST) underwent significantly more profound transcriptional remodeling compared to neuronal clusters (KCs, OPNs) (Figure 4D). Gene Ontology (GO) enrichment analysis of cell-type-specific differentially expressed genes revealed both conserved and divergent functional signatures between neurons and glia (Tables S9 and S10). Notably, a coordinated upregulation of stress-related terms, including ‘defense response’ and ‘immune system process’, was observed across both neuronal (KCs, OLCs) and glial (AST, EG, SG) populations, with OLCs additionally exhibiting specific enrichment for ‘cell killing’. Regarding lineage-specific alterations, SG uniquely exhibited enrichment for upregulated ‘maltose metabolic process’ genes, whereas other glial subtypes (AST, CG, EG) were characterized by the downregulation of ‘transporter activity’ and ‘voltage-gated ion channel activity’. Conversely, downregulated genes in neuronal populations (KCs, OLCs) predominantly mapped to synaptic signaling terms, including ‘extracellular ligand-gated monoatomic ion channel activity’ and ‘neurotransmitter receptor activity’. Collectively, glial populations exhibit more pronounced transcriptional perturbation characterized by metabolic and transport alterations, contrasting with the predominant downregulation of synaptic signaling components in neurons (Figure 4E).

### 3.4. Integrated Multi-Omics Analysis Suggests a Metabolic Shunt Towards the Pentose Phosphate Pathway in Glial Cells

**Figure 5:**
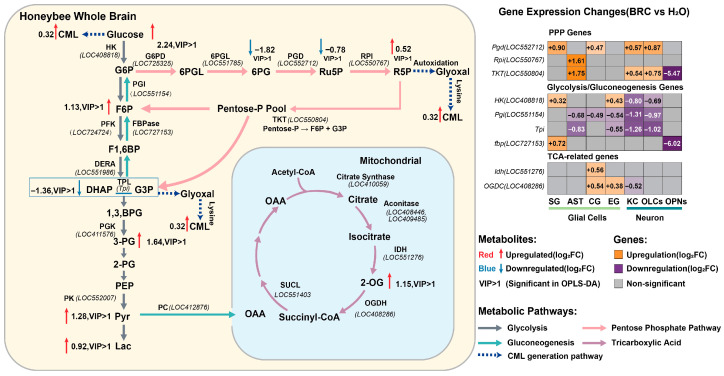
Integrated multi-omics analysis of the central metabolic network. The left panel displays the mapped changes in metabolites and enzymatic pathways within glycolysis, the pentose phosphate pathway (PPP), and the TCA cycle. Metabolites with red text/arrows indicate upregulation, while blue indicates downregulation (Log_2_FC, BRC vs. H_2_O). “VIP > 1” denotes metabolites with variable importance in projection scores greater than 1 in OPLS-DA. The dashed blue arrows represent the pathway leading to CML formation. The right panel shows a heatmap of differential gene expression (Log_2_FC) for key metabolic enzymes across distinct glial (SG, AST, CG, EG) and neuronal (KC, OLCs, OPNs) clusters. Orange indicates upregulation, purple indicates downregulation, and gray indicates non-significant changes (*p*_adj > 0.05).

To systematically explore the metabolic alterations associated with dopamine signaling, we mapped differentially expressed genes from single-nucleus RNA sequencing and differential metabolites from whole-brain LC-MS analysis onto the central metabolic network (Figure 5). At the metabolite level, elevated levels of key glycolytic and PPP intermediates were observed, including glucose, F6P, 3-PG, Pyr, Lac, and R5P (Figure 5, left panel). This pattern appears consistent with the cell-type-specific transcriptional profiles of rate-limiting enzymes. Specifically, the higher expression of *Rpi* parallels the accumulation of R5P. Notably, the gene encoding *Pgi*, which catalyzes the conversion of G6P to F6P, was downregulated in most cell types. This transcriptional suppression of *Pgi*, juxtaposed with the upregulation of PPP genes (*Pgd*, *Rpi*, *TKT*) in glial cells (Figure 5, right heatmap), could suggest a potential metabolic shunting mechanism where G6P is diverted towards the oxidative branch of the PPP, potentially supporting NADPH production and redox homeostasis. Furthermore, the integrated analysis points to a possible metabolic divergence between neurons and glia. Glial cells exhibited upregulation of glycolytic and PPP genes (e.g., *HK*, *Pgd*, *TKT*). In contrast, the reduced expression of *Tpi* in neurons might contribute to the accumulation of upstream triose phosphates and the observed elevation in methylglyoxal-derived advanced glycation end products, specifically CML (Figure 5, dashed lines). Concurrently, the upregulation of the TCA cycle intermediate 2-OG is consistent with the increased expression of *Idh* and OGDC in AST, suggesting that glial mitochondria may participate in replenishing TCA cycle intermediates. Collectively, these data suggest that dopamine receptor activation is associated with a metabolic profile characterized by enhanced glial aerobic glycolysis and PPP flux, alongside indicators of secondary glycative stress.

## 4. Discussion

Dopamine receptor activation in the honeybee brain was associated with a coordinated shift in central carbon metabolism, characterized by elevated glycolytic and pentose phosphate intermediates, increased levels of the advanced glycation end product N6-carboxymethyllysine, and cell-type-specific transcriptional changes. By integrating metabolomics with snRNA-seq, we show that this dopamine-associated metabolic reprogramming is not uniformly distributed across the brain but instead reflects enhanced glycolytic and PPP gene signatures, specifically *HK*, *Pgd*, and *TKT* in glial populations, against a backdrop of more heterogeneous neuronal responses.

Our study extends earlier work linking brain metabolism to social aggression and dopamine signaling in honeybees. Previous research indicated a link between neuromodulation, metabolic state, and behavior [6,7,42]. For example, Mustard et al. demonstrated that knockdown of the D1-like receptor *AmDop2* in mushroom bodies reduces walking and increases grooming and pausing [8]. Queen mandibular pheromone, a major social cue, also modulates brain dopamine levels and receptor gene expression [9]. However, it remained unclear whether dopamine signaling is associated with glycolytic reprogramming and which cell types are specifically affected. Our data show that activating D2-like receptors is associated with a glycolytic/pentose phosphate pathway-biased metabolome (e.g., accumulation of G6P, pyruvate, and R5P), with glycolytic gene signatures localized to glia rather than neurons. Specifically, our sncRNA-seq revealed that genes encoding rate-limiting enzymes such as *HK* were upregulated in SG and EG, contrasting with their downregulation in KCs. These findings advance our understanding of how glia-specific metabolic alteration may contribute to the behavioral effects of neuromodulation. This suggests that the observed metabolic shifts may reflect a common downstream metabolic convergence of dopamine signaling.

The metabolite profile and CML further suggest that dopamine receptor activation is associated with a coordinated accumulation of glucose and glycolytic intermediates, indicating a significant shift in the metabolic landscape and altered redox homeostasis. CML is a well-characterized advanced glycation end product formed through glycoxidation of glycated proteins, widely used as an integrated marker of chronic hyperglycemia and oxidative stress in human tissues [18,43]. In our dataset, increased CML co-occurs with elevated glucose, fructose, 3-phosphoglycerate, pyruvate, lactate, and selected PPP intermediates. This metabolic accumulation aligned with the transcriptional upregulation of key PPP genes, including *Pgd* and *TKT*, particularly in SG and AST populations. One parsimonious interpretation is that dopamine receptor activation is associated with elevated glucose and glycolytic metabolite levels in surface glia, coinciding with increased abundance of PPP intermediates potentially indicative of NADPH production to buffer oxidative stress, but at the cost of accumulating carbonyl species that may contribute to CML formation. Consistent with this model, dopamine uptake by striatal astroglia has been shown to markedly activate pentose phosphate pathway flux and reduce reactive oxygen species, and astrocytes with high glycolytic/PPP activity provide NADPH-dependent neuroprotection against oxidative and nitrosative stress [44,45,46], while glycolysis-derived methylglyoxal from triose phosphates is recognized as a major precursor of AGE adducts such as CML. Furthermore, the accumulation of CML indicates that the dicarbonyl/glycation stress associated with the high glycolysis and PPP metabolic rewiring is not fully neutralized, consistent with the CML-mediated oxidative stress and impaired cellular functions observed in multiple animal species [17,47,48]. In social insects, differences in energy metabolism, antioxidant defense, and somatic maintenance between highly active workers and long-lived reproductive individuals has been widely interpreted as an adaptive trade-off in the evolution of task specialization and lifespan [49,50,51]. Within this framework, we propose that the colony-level energetic benefits derived from intense foraging are likely prioritized by natural selection, even at the cost of accepting a certain degree of CML-related proteotoxicity and reduced individual longevity. Consequently, the observed CML accumulation can be regarded as a molecular marker of this metabolism–lifespan trade-off, rather than a purely pathological phenomenon.

The downregulation of several enzymes that connect deoxyribose-phosphate pools to glyceraldehyde-3-phosphate and reduced *Tpi* expression in neurons may reflect a compensatory attempt to limit triose phosphate accumulation and secondary glycation pressure in more vulnerable neuronal compartments. It is supported by evidence that spontaneous degradation of glyceraldehyde-3-phosphate and dihydroxyacetone phosphate is a dominant source of methylglyoxal and dicarbonyl stress [52,53]; that deficiencies in triosephosphate isomerase cause triose phosphate buildup, elevated methylglyoxal/AGE adducts [54,55]. Similarly, a discordance was observed between the downregulation of *Pgi* transcripts and the elevated levels of its product, fructose-6-phosphate. This transcriptional suppression could be interpreted as a dual homeostatic mechanism, whereby limiting *Pgi* abundance may favor the shunting of the glucose-6-phosphate pool into the upregulated pentose phosphate pathway for antioxidant defense, while simultaneously potentially functioning as a compensatory negative feedback response to the accumulation of downstream intermediates to prevent excessive glycolytic flux. This instance further highlights that steady-state metabolite levels reflect the net outcome of flux dynamics and allosteric regulation, which do not always linearly correlate with mRNA abundance [56]. Although our data do not directly measure flux, they align with the broader literature in which neuromodulator-associated shifts toward aerobic glycolysis and PPP activity are linked to behavioral plasticity and redox management in the brain [57,58,59].

In *Drosophila*, glial glycolysis and metabolite shuttling are recognized as key regulators of neuronal function, whereby glia sustain neuronal mitochondria and support memory by secreting glycolysis-derived lactate and alanine, or by upregulating compensatory fatty acid β-oxidation [60,61,62]. However, the conservation of such metabolic coupling in honeybees or other social insects remains unexplored. Our single-cell transcriptomic analysis of honeybee brains has revealed diverse glial and Kenyon cell subtypes. Building on this, we further found that surface glia exhibits the highest glycolysis scores and upregulation of key glycolytic and PPP genes (e.g., *HK*, *Pgd*, *fbp*) following dopamine receptor activation. Our observation therefore extends the mechanistic framework established in *Drosophila*, suggesting that the dopamine-associated bias toward glycolysis in barrier-associated glia may represent an evolutionarily conserved strategy for modulating the neuronal microenvironment and function. Crucially, in the context of social insects, this glia-derived metabolic support may be particularly vital for sustaining the high-energy demands of foraging flights and complex social behaviors. Foraging flight is a highly energy-demanding task. The chest flight muscles rely mainly on glucose and other hexose sugars as their fuel source [63,64]. The metabolic rate during flight is significantly higher than that during activities within the nest. Correspondingly, when worker bees shift from nurturing to foraging behavior, there are changes in the levels of dopamine in the brain and the expression of genes related to glucose metabolism and insulin signaling pathways [5,65,66,67]. Potentially by facilitating glucose mobilization and providing antioxidant protection via the PPP, this glial metabolic plasticity likely serves as a physiological buffer, ensuring neuronal stability during the metabolically taxing tasks of pollination and resource collection.

### Limitations of the Study

While this study offers a global view of brain metabolomics, several limitations should be acknowledged and addressed in future work. First, whole-brain metabolomics lacks spatial and cell-type resolution, which may obscure locally restricted metabolic alterations; integrating spatial metabolomics or emerging single-cell metabolomics could help overcome this constraint. Second, our data reflect steady-state metabolite abundances rather than dynamic reaction rates. While the concurrent upregulation of enzymes and intermediates strongly suggests pathway activation, we cannot rule out the possibility that metabolite accumulation results from downstream bottlenecks [68,69,70]. Future studies utilizing 13C-isotope tracing are necessary to definitively quantify metabolic flux and confirm the kinetics of this dopamine-associated reprogramming. Finally, the snRNA-seq approach primarily captures nuclear transcriptomes, potentially underrepresenting mitochondrial genes and subcellular compartmentalization [71]. Furthermore, biological interpretation of snRNA-seq data requires caution, as nuclear transcript abundance serves as a proxy for, but is not identical to, cytosolic mRNA levels or functional enzymatic activity. Consequently, the metabolic shifts observed in our single-nucleus data should be interpreted as changes in the ‘transcriptional potential’ of the metabolic network rather than direct measurements of cytosolic or mitochondrial reaction rates. Additionally, the whole-colony pharmacological approach, while essential for preserving natural foraging behaviors, precludes precise control of individual drug dosage. Developing sequencing methods that simultaneously profile cytoplasmic and organellar transcriptomes would provide a more integrated view of cellular metabolic logic.

## 5. Conclusions

In conclusion, this study presents an integrated multi-omics dataset characterizing the metabolic landscape associated with dopamine signaling in the honeybee brain. We observed that D2-like receptor activation coincides with a coordinated accumulation of central carbon metabolites and a glial-specific upregulation of glycolytic and pentose phosphate pathway genes. While the steady-state nature of our measurements limits definitive inferences regarding metabolic flux, these findings highlight a distinct glial metabolic signature associated with neuromodulation, providing a foundational resource for future investigations into insect brain bioenergetics.

## Data Availability

The raw and processed snRNA-seq data generated in this study have been deposited in the NCBI Gene Expression Omnibus (GEO) database under accession number GSE295726. The untargeted metabolism data and analysis code are available on Figshare (https://doi.org/10.6084/m9.figshare.30777629).

## Supplementary Materials

The following supporting information can be downloaded at: https://www.mdpi.com/article/10.3390/biology15020174/s1, Figure S1: Relative abundance of key metabolites in central energy metabolism pathways; Figure S2: Expression profiles of marker genes used for cell type annotation. Table S1: qPCR primer sequences; Table S2: Marker gene reference [23,72,73,74,75,76]; Table S3: Identification and intensity of metabolites in untargeted metabolomics. Table S4: Correlation analysis results and statistical metrics of targeted energy metabolism profiling in honeybee brains following bromocriptine treatment. Table S5: Detailed statistical analysis of targeted energy metabolism profiling in honeybee brains following bromocriptine treatment. Table S6: Enrichment analysis of metabolic pathways. Table S7: Counts and relative proportions of identified cell types in BRC-treated and control groups. Table S8: Cell-type-specific differential expression of key metabolic enzyme genes derived from snRNA-seq analysis. Table S9: The results of differentially expressed genes obtained from the analysis of all cells. Table S10: Functional enrichment analysis of cell-type-specific differentially expressed genes (|log_2_FC| > 1.5, min.pct > 0.1, *p*_adj < 0.05).

## Abbreviations

The following abbreviations are used in this manuscript:

LC–MS	Liquid chromatography–mass spectrometry
snRNA-seq	Single-nucleus RNA sequencing
CML	N6-carboxymethyllysine
PPP	Pentose phosphate pathway
DA	Dopamine
PGK1	Phosphoglycerate kinase-1
AGEs	Advanced glycation end products
PBS	Phosphate-buffered saline
BRC	Bromocriptine
QC	Quality control
PCA	Principal component analysis
PLS-DA	Partial least squares discriminant analysis
VIP	Variable importance in projection
FDR	False discovery rate
CCA	Canonical correlation analysis
KCs	Kenyon Cells
OLCs	Optic Lobe Cells
OPNs	Olfactory Projection Neurons
SG	Surface Glia
CG	Cortex Glia
EG	Ensheathing Glia
AST	Astrocyte-like Glia
IIS	Insulin/insulin-like growth factor signaling
TCA	Tricarboxylic acid
GO	Gene Ontology
OPLS-DA	Orthogonal partial least squares discriminant analysis
R5P	Ribose-5-phosphate
Glc	Glucose
F6P	Fructose-6-phosphate
Fru	Fructose
3-PG	3-phosphoglyceric acid
Pyr	Pyruvic acid
Lac	Lactic acid
DHAP	Dihydroxyacetone phosphate
Glu	Glutamate
Ru5P	Ribulose-5-phosphate
qPCR	Quantitative PCR
