# Supplementary material for: Metabolic Landscape and Cell-Type-Specific Transcriptional Signatures Associated with Dopamine Receptor Activation in the Honeybee Brain
Source: Biology (Basel). 2026 Jan 17;15(2):174. doi: 10.3390/biology15020174 (PMC12837817; doi:10.3390/biology15020174)
Supplement: Supplementary file 1 [file biology-15-00174-s001.zip › Table S1 qPCR primer sequences.pdf]

Table S1 qPCR primer sequences

Gene	seq
PKA-C1(LOC409791) F	TTTGCAGTCGCGGCATTGTT
PKA-C1(LOC409791) R	AGACTGGAGTTTGGTGGGGC
gapdh F	CACCTTCTGCAAAATTATGGCG
gapdh R	ACCTTTGCCAAGTCTAACTGTTAA
Dop3 F	TGGATCGAAATGCGGAACGG
Dop3 R	CCCTTTCTAATGCACTGTCGCT
