# Supplementary material for: Metabolic Landscape and Cell-Type-Specific Transcriptional Signatures Associated with Dopamine Receptor Activation in the Honeybee Brain
Source: Biology (Basel). 2026 Jan 17;15(2):174. doi: 10.3390/biology15020174 (PMC12837817; doi:10.3390/biology15020174)
Supplement: Supplementary file 1 [file biology-15-00174-s001.zip › Table S2 Marker gene reference.pdf]

Table S2 Marker gene reference.

Gene symbol	Gene ID in honeybee	Cell type	Citation	PMID	Title
repo	LOC410151	Glia	*Davie et al. 2018*	29909982	A Single-Cell Transcriptome Atlas of the Aging Drosophila Brain [72]
Vmat	LOC408517	Monoaminergic	*Allen,et al.2020*	32314735	A single-cell transcriptomic atlas of the adult Drosophila ventral nerve cord [73]
bdl	LOC551628	Glia			
e	LOC409109	Ensheathing			
fne	LOC410689	Neuron			
Gat-1B	Gat-1B	Astrocyte	*Li, Qiye et al. 2022*	35711063	A single-cell transcriptomic atlas tracking the neural basis of division of labour in an ant superorganism [74]
Gat-a	Gat-a	Astrocyte			
GlnS	GlnS	Astrocyte			
ldgf4	LOC413324	Ensheathing			

Mdr49	LOC551167	Surface
moody	LOC409159	Astrocyte
ninaB	LOC410521	photoreceptors
ninaC	LOC409937	photoreceptors
nSyb	LOC408465	Neuron
PKA-c1	LOC409791	Kenyon cells
Syt1	Syt1	Neuron
Tret1	*Tret1*	Surface
trio	LOC408594	Kenyon cells
trp	LOC724608	photoreceptors
vkg	LOC408551	Surface

wrap per	wrapper	Cortex			
zyd	LOC411976	Cortex			
grh	LOC411079	epithelial	*Hongjie Li,et al.2022*	35239393	Fly Cell Atlas: A single-nucleus transcriptomic atlas of the adult fruit fly [75]
PLCe	LOC408804	Kenyon cells	*Shota Suenami,et ai.2018*	30333766	Kenyon Cell Subtypes/Populations in the Honeybee Mushroom Bodies: Possible Function Based on Their Gene Expression Profiles, Differentiation, Possible Evolution, and Application of Genome Editing [76]
acj6	LOC410657	OPNs			
bsh	LOC724801	OLCs			
Eaat-1	LOC409919	OLCs			
eya	LOC412528	OLCs			
Hiscl1	*Hiscl1*	OLCs	*Zhang, Wenxin et al. 2022*	35800778	Single-cell transcriptomic analysis of honeybee brains identifies vitellogenin as caste differentiation-related factor [23]
hth	LOC552079	OLCs			
Lim1	LOC100577751	OLCs			
oaz	LOC413466	OPNs			

svp	LOC408872	OLCs
tsh	LOC102654448	OLCs
VACHT	LOC725064	Cholinergic
VGlut	LOC410752	Glutamatergic
mub	LOC408372	Kenyon cells
vsx1	LOC411315	OLCs
