# Supplementary figures and images for: Metabolic Landscape and Cell-Type-Specific Transcriptional Signatures Associated with Dopamine Receptor Activation in the Honeybee Brain
Source: Biology (Basel). 2026 Jan 17;15(2):174. doi: 10.3390/biology15020174 (PMC12837817; doi:10.3390/biology15020174)

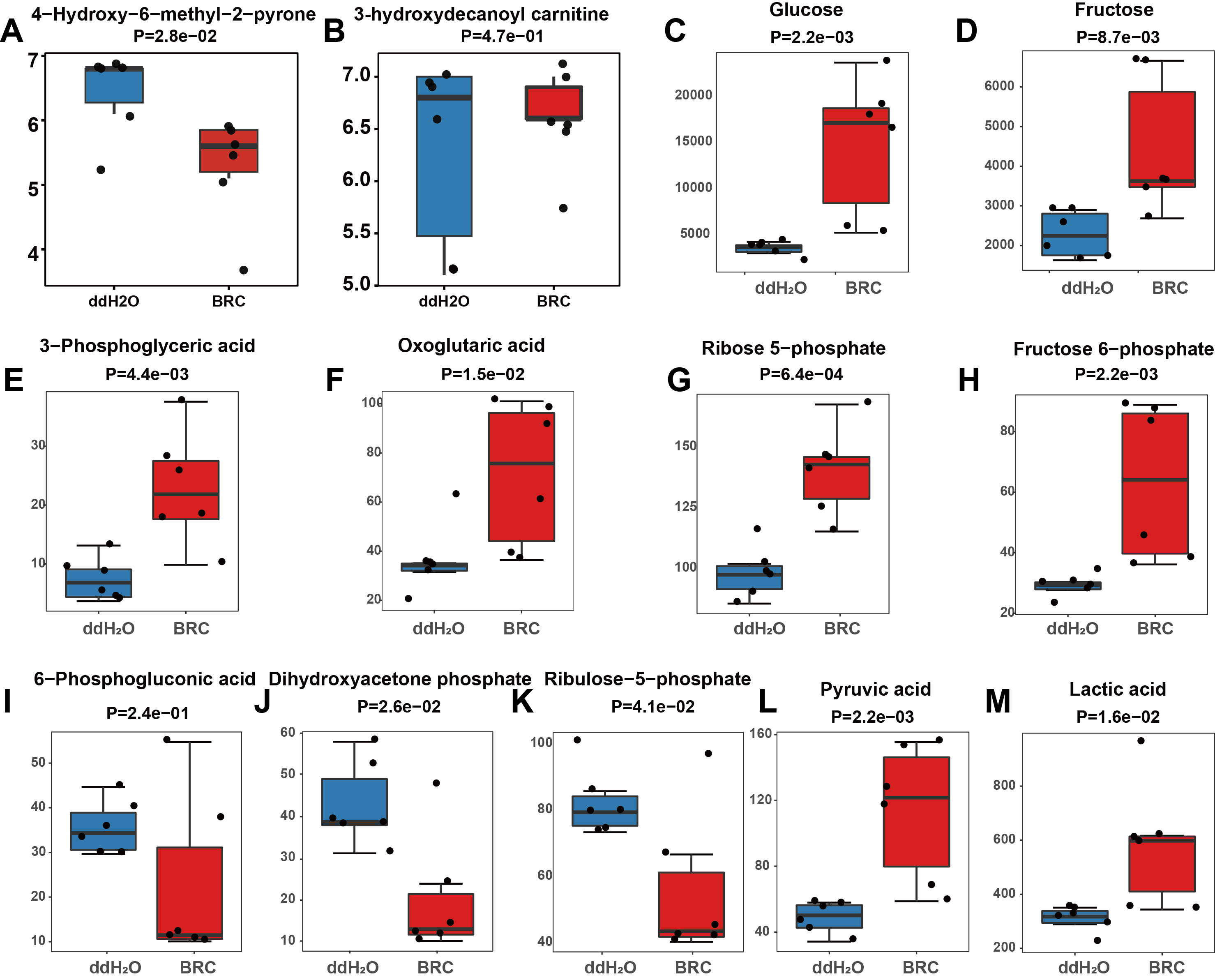

Supplement: Supplementary file 1 [file biology-15-00174-s001.zip › Figure S1.jpg]

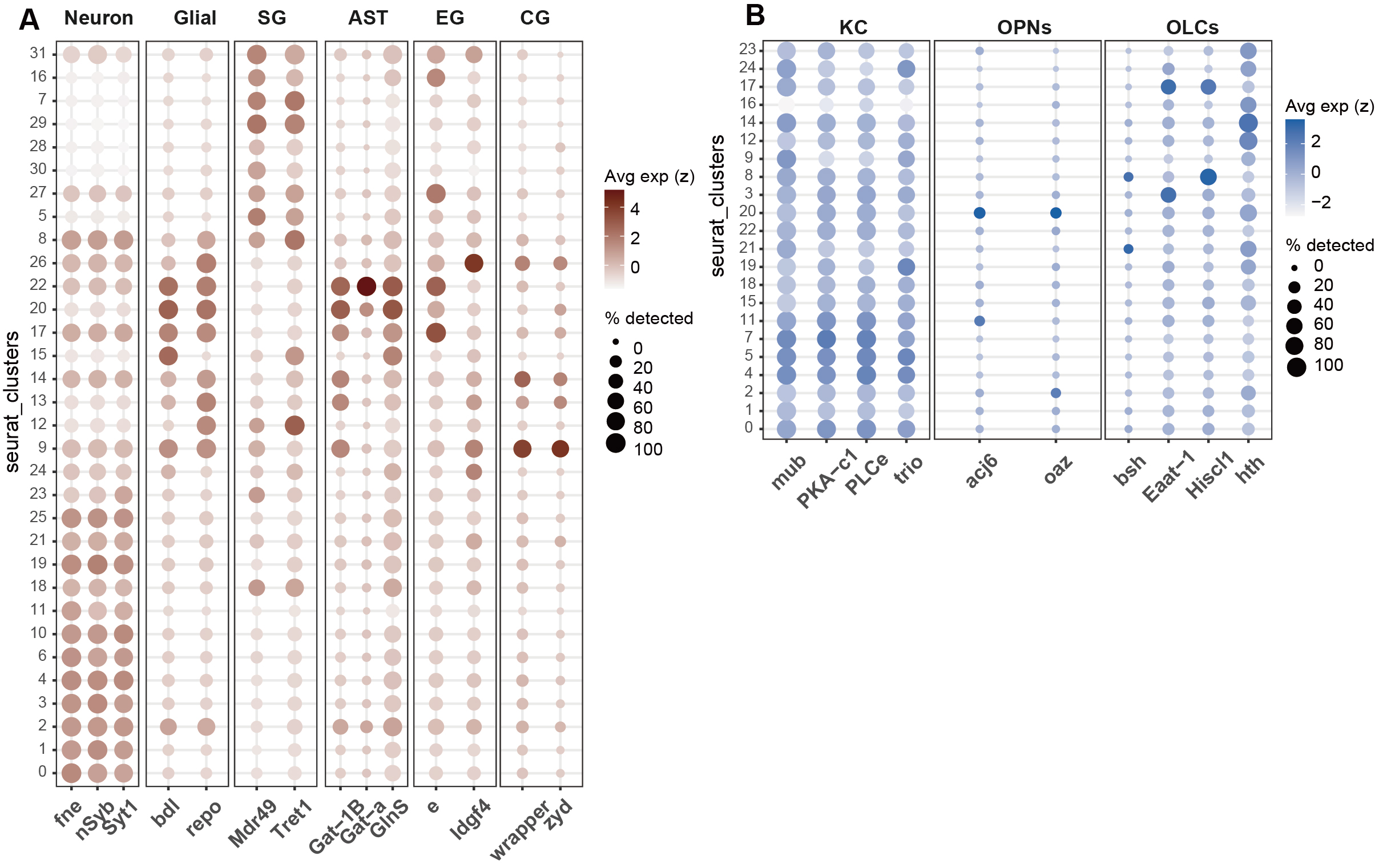

Supplement: Supplementary file 1 [file biology-15-00174-s001.zip › Figure S2.jpg]
